# Multiple variants of tick-borne encephalitis virus in voles, mice and ticks, the Netherlands, 2021 to 2023

**DOI:** 10.2807/1560-7917.ES.2025.30.4.2400247

**Published:** 2025-01-30

**Authors:** Emily L Pascoe, Julian W Bakker, Sara R Wijburg, Ankje de Vries, Hein Sprong, Matteo Marcantonio, Daniel Lang, Gerhard Dobler, Clara F Köhler, Helen J Esser, Constantianus JM Koenraadt

**Affiliations:** 1 Laboratory of Entomology, Wageningen University & Research, Wageningen, The Netherlands; 2 Conservation Genomics Research Unit, Research and Innovation Centre, Fondazione Edmund Mach, San Michele all’Adige, Italy; 3 Laboratory for Zoonoses and Environmental Microbiology, National Institute for Public Health and the Environment (RIVM), Bilthoven, The Netherlands; 4 Dutch Wildlife Health Centre, Faculty of Veterinary Medicine, University of Utrecht, Utrecht, The Netherlands; 5 Earth & Life Institute, University of Louvain (UCLouvain), Louvain-la-Neuve, Belgium; 6 Bundeswehr Institute of Microbiology, Munich, Germany; 7 Institute for Zoology, Parasitology Unit, University of Hohenheim, Stuttgart, Germany; 8 Department of Infectious Diseases and Tropical Medicine, University of Munich, Munich, Germany; 9 Wildlife Ecology & Conservation Group, Wageningen University & Research, Wageningen, The Netherlands; 10 LandLife Ecospatial Labs, Mezzocorona, Italy

**Keywords:** *Ixodes*, public health, surveillance, tick-borne encephalitis, tick-borne pathogens, zoonotic pathogens

## Abstract

**Background:**

Tick-borne encephalitis (TBE) can be a severe neurological disease. Identifying ecological factors that may facilitate tick-borne encephalitis virus (TBEV) circulation in the Netherlands could improve awareness and detection.

**Aim:**

We aimed to identify ecological factors affecting TBEV circulation in the Netherlands and to determine if there is sustained circulation and spread of the virus.

**Methods:**

Between June and September 2021, rodents and ticks from three previously TBEV-positive locations were tested for TBEV by PCR. We sequenced TBEV and compared the sequences with previous and subsequent sequences from the Netherlands and other countries to investigate the spread of TBEV-variants.

**Results:**

We captured 383 rodents, 928 feeding ticks and 1,571 questing *Ixodes* ticks and detected TBEV from six (three *Apodemus sylvaticus* and three *Clethrionomys glareolus*) (2.9%) of 206 tested rodents and two (0.9%) of 215 questing tick pools. Detection of TBEV was associated with questing tick density (Mann–Whitney U test  = 81.5; 95% confidence interval (CI): − 3.7–6.3 × 10^−5^; p = 0.05). Tick larvae (odds ratio (OR) = 9.0; 95% CI: 2.8–38.2; p < 0.01) and nymphs (OR = 3.8; 95% CI: 1.3–13.6; p < 0.01) were more frequent on *A. sylvaticus* than on *C. glareolus.* Sequence comparisons suggest multiple introductions and local circulation of TBEV but no spread among locations.

**Conclusion:**

Tick-borne encephalitis virus occurs in diverse woodlands in the Netherlands, posing a risk to those frequenting these areas. Surveillance for the early detection and monitoring of TBEV spread, along with public awareness campaigns on preventive measures, should continue. Recognition of TBE symptoms and supportive diagnostics should be made available nationwide.

**Key public health message**

**What did you want to address in this study and why?**

Tick-borne encephalitis (TBE) can be a severe neurological disease, caused by tick-borne encephalitis virus (TBEV) transmitted by ticks. In recent years, TBE has been diagnosed in new areas, including the Netherlands. We aimed to investigate the virus in rodents and ticks in the Netherlands to understand more about the virus circulation to guide future surveillance and detection efforts.

**What have we learnt from this study?**

We detected TBEV from six (2.9%) of 206 rodents and two (0.9%) of 215 pools of questing ticks in three locations in the Netherlands. Our data suggest that TBEV can remain and spread within different types of woodland.

**What are the implications of your findings for public health?**

Our findings indicate that TBEV is present throughout the Netherlands. Tick bite prevention campaigns should highlight TBE as a potential risk. Continued surveillance to detect and monitor TBEV can support public health strategies (e.g. vaccination campaigns), but awareness and availability of diagnostics for TBE among health professionals should be made nationwide and not only in locations where TBEV circulation is confirmed.

## Introduction

Reports of human tick-borne encephalitis (TBE) have been increasing throughout Europe [1,2], and the causative virus, tick-borne encephalitis virus (TBEV), continues to be detected in new locations [3]. Within the last two decades, TBEV has been detected for the first time in at least five countries in Europe, including the Netherlands [4,5].

Predictive models aid in focussing pathogen detection and surveillance efforts which are necessary for timely responses [6]. Prior to detection in the Netherlands, some previous models did not consider the country climatically suitable for the circulation of TBEV [7,8]. Since 2015, TBEV has been confirmed in 10 locations in the Netherlands [4,9-11]. Climate is not the only factor in predicting presence of TBEV, and in-depth ecological studies are necessary to determine abiotic and biotic factors that may promote TBEV circulation. These data would guide more effective surveillance and detection efforts.

The objectives of this study were to: (i) determine which ecological factors (i.e. habitat type, rodent and tick community characteristics) are associated with TBEV; (ii) determine if there is sustained circulation and spread of TBEV, within and among locations by comparing sequences within the Netherlands and comparing the Dutch sequences with those from other countries across multiple years. These data were combined with ongoing surveillance efforts that aim to continuously monitor TBEV circulation in the Netherlands, and link findings with the detection of human cases (and vice versa).

## Methods

### Sampling locations

**Figure 1:**
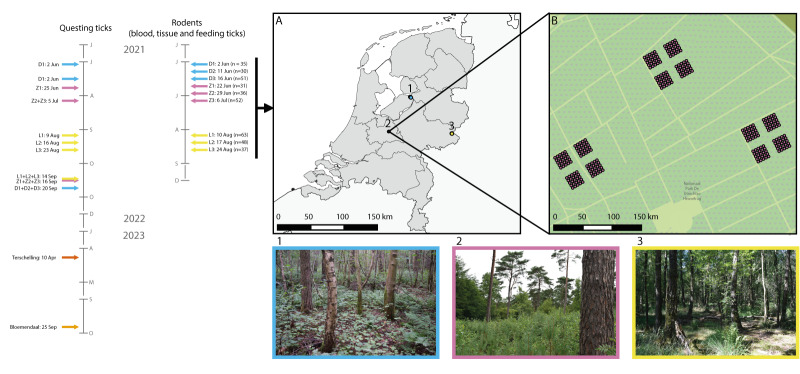
Timeline and geographic location of rodent and tick sampling in a survey of tick-borne encephalitis virus, the Netherlands, 2021–2023

Specimens were collected between June and September 2021 from three locations in the Netherlands (Dronten, Zeist and Oost Gelre) (Figure 1) where TBEV had been detected ≤ 2 years earlier [9-11]. Locations, plots and trapping grids therein varied considerably with respect to the dominant vegetation (tree and understorey plants) present, as determined by vegetation surveys conducted in August 2022. Dronten was mostly characterised by dense, highly mixed woodland (coniferous and broadleaf species), the habitat at Zeist was mainly composed of coniferous woodland and at Oost Gelre, small patches of broadleaf woodland were surrounded by water. Further details of vegetation species identified at each location, plot and grid are presented in Supplementary Table S1.

D: Dronten; L: Oost Gelre; Z: Zeist.

Panel A. Sampling in three locations: 1) Dronten, 2) Zeist and 3) Oost Gelre.

Panel B. Three plots at least 350 m apart were established at each location. At each plot, 100 live rodent traps were placed in four 5 × 5 square grids. Each trap is depicted as a pink circle on the map.

Map was created using EPSG: 32431 (https://epsg.io/). Shapefile from https://diva-gis.org/data.html. Inset map data copyrighted by OpenStreetMap contributors and available from (https://www.openstreetmap.org).

### Rodent sampling

Rodent sampling was performed between June and August 2021. To maximise the possibility of detecting TBEV, while minimising the likelihood of recapturing or sampling the same rodent individuals, each sampling location was divided into three plots of 1.44 ha, 350–1,550 m apart. At each plot, 100 live traps (Heslinga Traps, Groningen, the Netherlands) were arranged in four grids (Figure 1). Rodent trapping was performed once at each plot as previously described [12]. For animal welfare reasons, all captured animals were released within 12 h. Species, sex and reproductive status were recorded for all captured individuals. Except for plot Z1, due to the time restrictions, we could not collect specimens from all eligible rodents and used simple random sampling of rodents until the 12 h time limit was reached. Blood and ear tissue sampling was performed as previously described for rodents that weighed ≥ 20 g, appeared in good health and were not evidently pregnant or nursing [12]. Ear tissue was immediately stored in 90% ethanol. The number of tick larvae (when > 20, the number was approximated) and nymphs feeding on each rodent were noted for all plots, except for D1. All nymphs and ca 5–20 larvae were collected (favouring those that were engorged and/or close to feeding nymphs). These feeding ticks are henceforth referred to as body ticks, whereas ear ticks were collected exclusively from biopsied ear tissue (as described in Detection of tick-borne encephalitis virus from tick and rodent specimens). All specimens were stored at −80°C within 24 h.

### Questing tick community

We sampled questing ticks between June and September 2021. To collect questing ticks from the vegetation, tick dragging was conducted by dragging a 1 m wide cloth at each plot along a 240 m^2^ transect, i.e. from one end of the plot to the other (120 m), and back along a parallel transect. In addition, we dragged along the length of the plot on any bordering recreational trails, as a proxy for a risk of human exposure. Dragging was performed at each plot on two occasions; within 6 days of rodent sampling (considered the same sampling session) and in the second half of September along approximately the same transects. Prior to extraction of total nucleic acids (TNA), questing ticks were grouped into pools of 1–16 ticks, according to transect and life stage, except for one pool from Z1 which contained both a nymph and larvae. A table detailing each pool of ticks is available in Supplementary Table S2.

### Detection of tick-borne encephalitis virus from tick and rodent specimens

Ear tissue was removed from ethanol, dried, and any ear ticks feeding on the tissue were collected. A visible blood meal or engorgement was noted for each body and ear tick before individual TNA extraction. Extraction of TNA and subsequent detection of TBEV-RNA from all specimens from rodents (ear tissue, ear ticks, dried blood spot (DBS), body ticks) and questing ticks was performed as previously described [12]. Sequences were generated from a TBEV-positive specimen (with the lowest quantification cycle (Cq) score) from each of the three locations using random primers (Invitrogen, Thermo Fisher Scientific, Waltham, the United States (US)) for reverse transcription using Superscript IV (Invitrogen), after which PCR was carried out with primer pairs for overlapping fragments [13]. Fragments were pooled and Nanopore sequencing was performed using the Native Barcoding Kit (Oxford Nanopore Technologies, Oxford, the United Kingdom (UK); EXP-NBD104 and SQK-LSK109) on a FLO-MIN106D Flow Cell.

### Phylogenetic analyses of tick-borne encephalitis virus

Phylogenetic analyses were performed as previously described [9], using MEGA software version 11.0.13 (https://www.megasoftware.net/), on 13 sequences from the Netherlands: three from the current study, eight published sequences and two obtained through surveillance efforts associated with autochthonous human TBE cases in the Netherlands in 2023 (Figure 1). In April 2023, a nymph was collected from a TBE patient who had been bitten by a tick in Terschelling, and in September 2023 tick dragging was performed in Bloemendaal after a human TBE case was detected in the area. The 10 most closely matching sequences published in GenBank (https://www.ncbi.nlm.nih.gov/genbank/) based on the BLAST Max Score for each of these 13 sequences were included (some of the sequences among the 13 BLAST results were duplicated). A multiple sequence alignment was performed using the MUSCLE algorithm version 3.8.31 [14]. The maximum likelihood method and general time reversible model with a gamma distribution and invariant sites was used, and the highest log likelihood tree following 1,000 bootstrap iterations was visualised [15].

### Statistical analyses

To quantify species evenness of the rodent community at each plot, Pielou’s evenness indices were calculated [16]. Questing tick density was calculated for each plot for larvae, nymphs, adult males, adult females and total ticks as the number of ticks collected by dragging per 100 m^2^ dragging transect. Fisher’s exact tests were used to test for associations between ecological factors (i.e. habitat type, rodent and tick community characteristics) and detection of TBEV. Specifically, we tested for associations between (i) rodent species (the wood mouse *Apodemus sylvaticus* and the bank vole *Clethrionomys glareolus*) and tick larvae and nymph burden, and detection of TBEV from specimens; (ii) visible blood meal and detection of TBEV in a feeding tick and (iii) habitat type and detection of TBEV from specimens. Habitat type (summarised as broadleaf or mixed woodland) was characterised based on the vegetation survey data. Mann–Whitney U tests were performed to test for an association between the detection of TBEV from questing ticks or rodent specimens at a plot during a sampling session and the total number of rodents captured, Pielou’s evenness indices, percentage of male rodents, rodent tick burdens (number of feeding larvae, nymphs and total per animal) and questing tick density (for each life stage and total) during a sampling session. Mann–Whitney U tests were used to compare sequence similarities for Dronten (two from 2020 and one from the current study) and Zeist (one from 2016, one from 2018 and one from the current study), from the same vs different locations.

To infer TBEV circulation dynamics, we tested for over-representation of TBEV variants in Europe [17]. Sequence records of TBEV with ≥ 8,000 nt were downloaded from GenBank (n = 464). The awk version 5.1.0 Unix programme [18] was used to extract records with information on the country of origin. Sequences from non-European countries were excluded. The resulting 202 sequences were clustered into TBEV variants at a threshold of ≥ 99% similarity (n = 154) using CD-HIT [19]. We used a multinomial test in which the number of trials corresponded to the number of sequences from each country and the probability of obtaining a TBEV variant from the respective country was equal for each of the 154 that were identified. The Monte Carlo method, with 10,000 simulations, was used to estimate the probability that the maximum number of TBEV variants per country in a random realisation from the multinomial test resulted in a number of TBEV variants equal to or exceeding the number observed. Thus, p ≤ 0.05 indicated that the observed number of variants for a country was lower than expected by chance, and therefore over-representation of one or more TBEV-variants, while p > 0.05 suggested that the observed number of TBEV-variants in the country did not deviate from random simulations. Statistical analyses were performed in R version 4.3.1 [20].

## Results

### Rodent community

**Figure 2:**
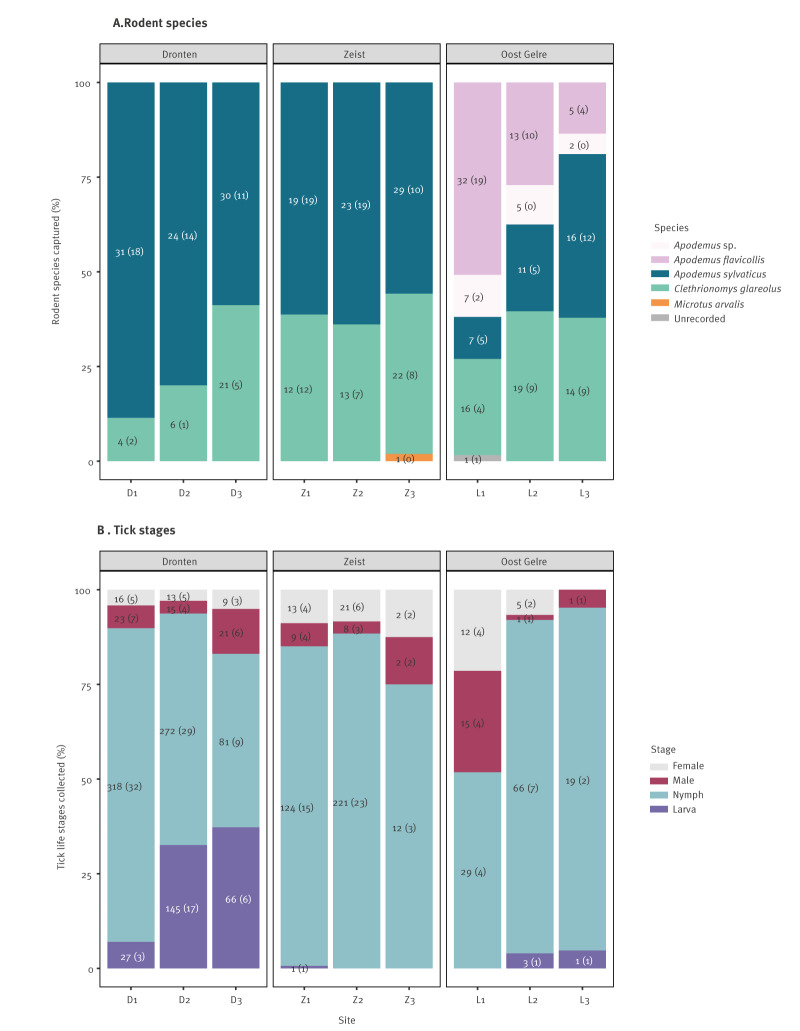
Rodents (n  =  383) and questing ticks (n  =  1,571) captured in a survey of tick-borne encephalitis virus, by rodent species and tick stage, the Netherlands, June–September 2021

In 2021, we captured 383 rodents: 190 *A. sylvaticus*, 127 *C. glareolus*, 50 yellow-necked mice (*Apodemus flavicollis*), one common vole (*Microtus arvalis*), 14 *Apodemus* (species could not be identified) and one rodent with genus not recorded (Figure 2). Although *Apodemus* was the most abundant genus, *A. flavicollis* was only found in Oost Gelre and *M. arvalis* was captured from one Zeist plot (Figure 2). In total, 228 rodents were adults, and 42.0–50.7% per location were female (total number of females: 182). Additional details on sex, reproductive status and tick burden per plot are presented in Supplementary Table S3.

D: Dronten; L: Oost Gelre; sp.: species; Z: Zeist.

Panel A. Numbers of rodent species captured are presented in the bars, numbers of rodents from which samples were taken are presented in the brackets.

Panel B. Numbers of tick stages collected are presented in the bars, numbers of pools (grouped by dragging transect and life stage) tested for tick-borne encephalitis virus are presented in the brackets. At site Z1, one pool contained both a nymph and nine larvae, contributing to two bar segments. Thus, the total number of pools (n = 23) is less than the sum of brackets (n = 24) for this plot.

A total of 205 rodents were checked for ticks, of which 176 (85.9%) were infested with larvae: 95 (96.0%) of 99 *A. sylvaticus*, 28 (84.8%) of 33 *A. flavicollis*, 52 (72.2%) of 72 *C. glareolus* and one rodent with genus not recorded. Presence of tick larvae was more common on *A. sylvaticus* (odds ratio (OR) = 9.0; 95% confidence interval (CI): 2.8–38.2; p < 0.01) and median larval burden was 25 (range: 2–130), compared with *C. glareolus*, which had a median larval burden of 3.5 (range:  1–67). Nymphs were only present on two rodent species and on individuals that also harboured larvae: 22 (22.2%) of 99 *A. sylvaticus* and 5 (6.9%) of 72 *C. glareolus.* Likewise, nymphs were significantly more common on *A. sylvaticus* (OR = 3.8; 95% CI: 1.3–13.6; p < 0.01) compared with *C. glareolus*. The number of nymphs varied from 1 to 4 for both species. By location, 32 of 35 rodents from Dronten had larvae and four had nymphs, 74 (98.7%) of 75 rodents from Zeist had larvae and 18 (24.0%) had nymphs, and 70 (73.7%) of 95 rodents from Oost Gelre had larvae and five (5.3%) had nymphs.

### Tick community

We collected 1,571 questing and 928 feeding ticks, all of which were of the genus *Ixodes*. Despite temporal differences in questing tick sampling, questing nymphs were the most abundant life stage (n  =  1,142) at all locations, but proportions of all life stages varied (Figure 2). The questing ticks from the three locations were tested for TBEV in 215 pools separated by plot and life stage, except for one tick pool from Z1 which contained one larva and nine nymphs.

### Detection of tick-borne encephalitis virus

We tested specimens from 206 rodents for TBEV-RNA: 113 *A. sylvaticus*, 33 *A. flavicollis*, 57 *C. glareolus*, two unidentified *Apodemus* spp. and one individual with unrecorded species. From these rodents, ear tissue from 79 individuals, DBS from 112 individuals, 49 ear ticks from 25 individuals (1–7 ticks per rodent) and 879 body ticks from 195 individuals (1–17 ticks per rodent) were tested. The number of specimen types per individual varied as not all rodents met the criteria for blood and tissue sampling (in some instances DBS were collected from rodents that did not meet the sampling criteria, e.g. from the skin site where ticks were removed). Tick-borne encephalitis virus was detected from six (2.9%) rodents: from three *A. sylvaticus* (three ear specimens, one DBS, one body tick; n  =  5) and three *C. glareolus* (two ear specimens, one DBS, one ear tick, 13 body ticks; n  =  17) (Table). There was no difference in detection of TBEV among all specimens from these two species (OR = 2.0; 95% CI: 0.3–15.2; p = 0.41). Of the TBEV-positive body ticks, four were from a rodent ineligible for ear or DBS sampling. There was visible evidence of a blood meal in 9 of 15 positive feeding larvae, but this was not significantly associated with TBEV detection (OR = 4.0; 95% CI: 0.8–23.1; p = 0.08). No nymphs were found on the six TBEV-positive rodents.

**Table:** Specimens from rodents that tested positive for tick-borne encephalitis virus, the Netherlands, 2021 (n  =  6)

Location	Plot ID	Species	Sex	Breeding status	Larvae (n)	Ear tissue	Ear tick		Body tick		Dried blood spot
							TBEV positive	All	TBEV positive	All	
Dronten	D1	*A. sylvaticus*	Male	Adult	NC	Pos	0	1^b^	1^b^	5^c^	Pos
Dronten	D3	*A. sylvaticus*	Female	Adult	15	Pos	NA	NA	0	6^c^	Neg
Zeist	Z1	*C. glareolus*	Male	Adult	60	Pos^a^	NA	NA	4^d^	5	Pos
Zeist	Z1	*A. sylvaticus*	Male	Adult	28	Pos	0	1	0	4	Neg
Zeist	Z2	*C. glareolus*	Male	Sub-adult	24	NA	NA	NA	4^d^	5	NA
Oost Gelre	L1	*C. glareolus*	Male	Adult	7	Pos^a^	1^b^	2^c^	5^b^	5	NA

*A*.: *Apodemus*; *C.: Clethrionomys*; ID: identification code; NA:  specimen not available, e.g. because animal was ineligible for sampling or ear ticks were not present; NC: not counted; Neg: negative; Pos: positive.

^a^ Specimen sequenced.

^b^ One tick with some degree of blood meal or engorgement.

^c^ Two ticks with some degree of blood meal or engorgement.

^d^ Three ticks with some degree of blood meal or engorgement.

Only larval ticks were found.

We detected TBEV from 2 (0.9%) of 215 questing tick pools. Both positive pools were from Dronten: one pool of nymphs from D3 (wet woodland dominated by willows; *Salix* spp.) collected in June and one pool of nymphs from a road verge frequented by hikers and cyclists bordering plot D2 (mixed broadleaf and coniferous woodland), collected in September.

### Ecological factors associated with detection of tick-borne encephalitis virus

Tick-borne encephalitis virus RNA was detected at all three locations: one plot at Oost Gelre, two at Zeist and all three plots at Dronten. Specifically, TBEV was detected where habitat was characterised as: dense, mixed, broadleaf and coniferous woodland; wet woodland dominated by goat willow (*Salix caprea*); thinned stands of Scots pine (*Pinus sylvestris*) with a lower layer of silver birch (*Betula pendula*); and a pedunculate oak dominant woodland (*Quercus robur*). There was no significant difference in TBEV detection between broadleaf or mixed woodlands (OR = 2.2; 95% CI: 2.1 × 10^−2^–234.2; p = 1).

Average density of questing males and total questing ticks at plots, where TBEV was detected, was 2.7 ticks per 100 m^2^ and 40.4 ticks per 100 m^2^, respectively, compared with 0.9 ticks per 100 m^2^ and 26.1 ticks per 100 m^2^, respectively, at plots where TBEV was not detected. Density of adult males (U = 79.5; 95% CI: −2.9¬ − 5.7 × 10^−5^; p = 0.04) and total tick density (U = 81.5; 95% CI: −3.7–6.3 × 10^−5^; p = 0.05) were associated with detection of TBEV from a plot, but questing larvae (U = 140; 95% CI: −1.6 × 10^−5^–3.0 × 10^−5^; p = 0.88), nymphs (U = 86.5; 95% CI: −29.8–0.4; p = 0.08) and adult females (U = 109.5; 95% CI: −1.7–0.4; p = 0.34) were not. We found no association between the detection of TBEV from a plot and the total number of rodents captured (U = 13.0; 95% CI:  −26.0–22.0; p = 0.37), rodent species evenness (U = 9.0; 95% CI: −0.3–0.5; p = 1), nor percentage of male rodents (U = 4.5; 95% CI:  −22.4–8.3; p = 0.30). There was also no association between the detection of TBEV and number of larvae (U = 1.0; 95% CI: −54.7–0.2; p = 0.07), number of nymphs (U = 5.0; 95% CI:  −0.7–0.1; p = 0.55), nor total number of ticks (U = 1.0; 95% CI: −54.7–0.2; p = 0.07) on rodents.

### Sequences and variants of tick-bone encephalitis virus

**Figure 3:**
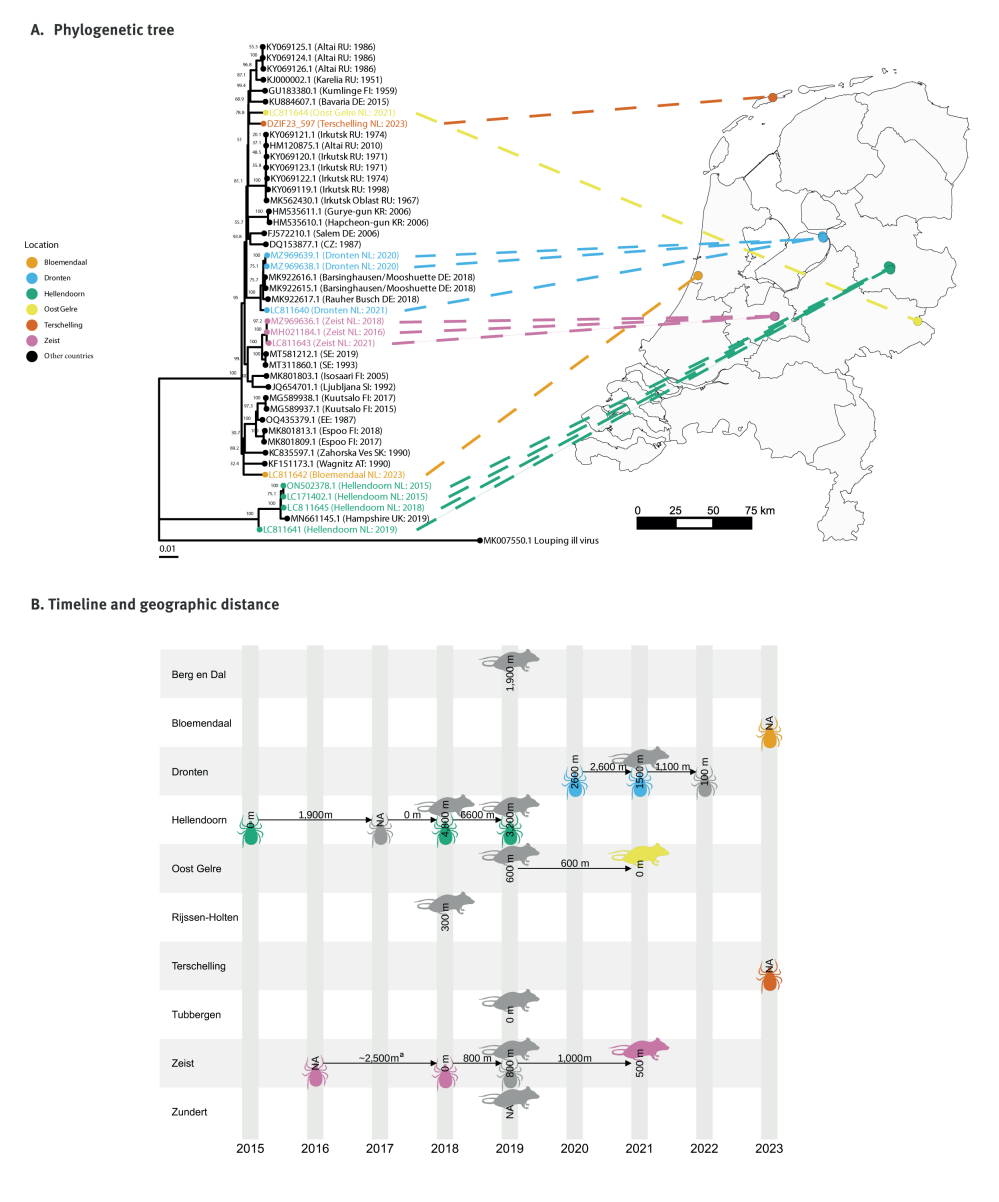
Maximum likelihood phylogenetic tree of sequences of tick-borne encephalitis virus, the Netherlands, 2015–2023 (n  =  13) and sequences from GenBank, 1951–2019 (n = 32) and timeline and geographic location of rodents and ticks positive for tick-borne encephalitis virus, the Netherlands, 2015–2023 (n  =  13)

AT: Austria; CZ: Czechia; DE: Germany; EE: Estonia; FI: Finland; ID: identification code; KR: South Korea; NA: not possible to estimate a distance; NL: the Netherlands; RU: Russia; SE: Sweden; SI: Slovenia; SK: Slovakia; TBEV: tick-borne encephalitis virus; UK: the United Kingdom; 0 m:  several TBEV-positive specimens were collected from the same coordinate or plot.

We obtained TBEV sequences from specimens of *C. glareolus* ear tissue from Zeist (accession: LC811643) and Oost Gelre (LC811644) and from a pool of questing nymphs from Dronten (LC811640). Within the phylogenetic tree, the Oost Gelre sequence was most similar (98.46%) to a sequence from the Altai Republic of Russia (KY069125.1) and to the 2023 sequence from Terschelling island (DZIF23_597) (Figure 3). Some sequences were available from Dronten (two from 2020 and one from this study) and Zeist (one from 2016, one from 2018 and one from this study), and sequences from the same location were significantly more similar than those between the two locations (U = 54.0; 95% CI: 1.7–2.1; p < 0.01). The sequence from Dronten in our study was 99.48% and 99.51% similar to those obtained in 2020 (MZ969639.1 and MZ969638.1, respectively). The sequence from Zeist obtained in this study was 99.85% and 99.88% similar to sequences collected there in 2018 (MZ969636.1) and 2016 (MH021184.1), respectively (Figure 3). By comparison, the three sequences obtained from each location were no more than 98.13% similar to one another. The Terschelling and Bloemendaal locations detected in 2023 may represent introductions of new variants to the Netherlands: the Terschelling sequence was most similar (98.55%) to two sequences from Russia (KY069124.1 and KY069125.1) and 98.46% similar to Oost Gelre, and the Bloemendaal (LC811642) sequence was 98.09% similar to a sequence from Estonia (OQ435379.1) and 97.80% similar to any other Dutch sequence (i.e. from Terschelling). Despite variation in sequences among locations in the Netherlands, we found significant over-representation of one or more TBEV-variants across the country among the 154 variants identified throughout Europe (p < 0.01). A full list of all sequences used in the analyses is available in Supplementary Table S4, and the subsequent results for each European country are shown in Supplementary Table S5. When we collated coordinates of TBEV-positive specimens in the current study with those from previous studies in the Netherlands [4,9,10], we found that within and between calendar years TBEV-positive specimens could be located 0– ≥ 6,600 m apart at a location (municipality) (Figure 3).

Panel A. Sequences from the Netherlands were obtained from Bloemendaal, Dronten, Hellendoorn, Oost Gelre, Terschelling and Zeist, the other sequences from GenBank (https://www.ncbi.nlm.nih.gov/genbank/). Louping ill virus was used as an outgroup. Specimen ID or accession numbers are indicated for each sequence, with location (if known), country code and collection year in brackets. Numbers next to each branch indicate the percentage of trees resulting from bootstrapping based on 1,000 pseudoreplicate datasets for which the associated taxa clustered together. The scale bar associated with the phylogenetic tree represents the percentage of genetic variation along tree branches. The geographic locations where each sequenced specimen was collected within the Netherlands is indicated, when coordinates were not available, the location was approximated.

Panel B. Timeline of detection of TBEV from ticks and rodents in municipalities in the Netherlands, acquired as part of the current study, published studies or ongoing surveillance. The maximum known distance between two positive specimens is indicated for each year as well as between years. Pictograms depict whether positive specimens were from rodents or ticks during a given year, and those that are in colour indicate that a sample was sequenced.

^a^ Exact location unknown so distance estimated.

## Discussion

We present evidence of at least six TBEV introduction events in the Netherlands, including two locations identified in 2023 (Bloemendaal and Terschelling). We detected TBEV-RNA in rodents and ticks (including those feeding on a host) in three diverse woodland locations, with high sequence similarity within locations across years. Together, these findings suggest that following introduction, TBEV circulation can be sustained between vectors and reservoir hosts in the Netherlands, particularly when tick densities are high. While local spread may occur, we did not observe the spread of TBEV-variants among locations.

Our data corroborate that tick-to-tick transmission of TBEV via blood feeding on a host is not always successful [21], and presence (or absence) of TBEV-RNA in feeding ticks does not necessarily indicate the infection status of the host. However, testing feeding ticks could be an option to detect TBEV if tissue or blood samples are not available. Not all TBEV-positive feeding larvae were visibly engorged, suggesting that either low volumes of blood are sufficient for ticks to test positive or that larvae had become infected via transovarial transmission [22,23]. Transovarial transmission of TBEV in ticks has been confirmed experimentally [22], but its role in TBEV ecology has been debated [23]. Moreover, we did not find any nymphs feeding on TBEV-positive rodents, so co-feeding transmission to these larvae may be less likely. The relative role of TBEV transmission among ticks in nature via transovarial transmission, co-feeding and from direct feeding on a systemically infected host is unclear.

We detected TBEV from relatively more samples and feeding ticks from *C. glareolus* (n  =  17) than from *A. sylvaticus* (n  =  5), yet ticks were less commonly found on *C. glareolus* (but the numbers of ticks on infested individuals did not differ between species). As we detected TBEV from only three *C. glareolus* and three *A. sylvaticus*, we cannot determine if *C. glareolus* are more efficient for virus transmission to ticks via systemic infection or co-feeding. *Clethrionomys glareolus* may be important in the Dutch TBEV sylvatic cycle, as they are elsewhere in Europe [24-26]. However, the long-term role of *C. glareolus* is unclear, as they can develop immune-mediated resistance to tick feeding which may limit overall virus transmission [21,27,28]. We found no association between rodent community characteristics (total numbers, sex ratio and species evenness) and detection of TBEV, but the specific role of different rodent species appears to be complex, and further data (e.g. natural viraemia, tick feeding success) from multiple rodent species are necessary to understand their relative role in TBEV circulation.

We detected TBEV in varied habitats in which species composition of rodents and therefore possibly reservoir hosts differed, adding to evidence that TBEV can circulate in a broad range of environments [29-31]. Complex interactions between tick and rodent population dynamics, as well as climate, habitat and other tick feeding hosts, such as ungulates are likely the main drivers of TBEV presence and persistence [30,32-35]. While we observed that TBEV presence is associated with high densities of questing ticks, more data are required to better tease apart such complex interactions and to predict areas most at risk. Detecting TBEV within sylvatic cycles is challenging; thousands of ticks may need to be tested to find a single positive pool [9] and there are ethical and logistical aspects to testing wild animals. Combining these data sources with environmental information on a larger scale is needed to improve monitoring and surveillance.

The similarity of sequences from Oost Gelre and Bloemendaal to sequences from Estonia (BLAST results) is compelling. Given the spatial and temporal distance of these sequences, it is possible that these TBEV-variants arrived in the Netherlands via intermediate locations. Migratory birds or bats may be involved in TBEV introductions, either by serving as a vehicle for infected ticks, or by transmitting the virus to ticks once in the country [36-39]. Indeed, the sequence locations in the Netherlands and Estonia lie along the migratory routes of some bird and bat species that are potential TBEV reservoirs or disseminators [40-43]. In addition, Terschelling is a small island located in the Wadden Sea, and the virus may have been introduced there by flight. With this in mind, it could be prudent to focus efforts to detect new TBEV hotspots along such flyways across Europe.

Collectively, data indicate that TBEV can be present in ticks or rodents in the Netherlands, with widespread occurrences in wooded areas (e.g. TBEV samples have been collected approximately  12.4 km apart within National Park Sallandse Heuvelrug, in the municipalities Hellendoorn and Rijssen-Holten), over which habitat can vary substantially [4,9,10]. However, without fine spatial scale sampling or genetic information pertaining to all positive specimens, we cannot determine if TBEV occurs in a single large focus or in multiple micro-foci within all locations [36,44]. Regardless, data indicate a potentially long season across a wide area of circulation and risk to human health. Vigilance for tick bites should be recommended year-round and across all recreational areas in the Netherlands.

Despite being collected no more than 5 years apart, sequence similarities at foci (99.48–99.88%) were comparable to other countries where TBEV has been circulating for decades, e.g. Kumlinge (Finland) where sequences collected 44 years apart were 99.7% similar [39]. Our analyses suggest that multiple TBEV-variants circulate in the Netherlands. We note, however, that this may be subject to sampling bias (related to sampling locations and intensity).

The risk of infection with tick-borne pathogens is reduced by avoiding tick bites and promptly removing ticks from the body. Information campaigns for avoiding tick bites already exist in the country and are widespread (due to the risk of Lyme disease) but should include TBE among the possible risks associated with tick bites in the Netherlands.

## Conclusion

We demonstrate that TBEV is present in *C. glareolus*, *A. sylvaticus* and ticks in different locations in the Netherlands. While national vaccination campaigns for TBEV are not yet warranted, vaccination of high-risk groups (i.e. those frequenting woodland) has recently been advised by the National Health counsel. We thus advise awareness of TBEV and provisions of supportive diagnostics for health professionals nationwide, rather than only in locations where TBEV circulation is confirmed so that prompt diagnosis can enable more effective supportive care of TBE patients. Surveillance efforts should continue nationwide for the early detection and monitoring of TBEV spread, supporting timely interventions and public health strategies.

## Supplementary Data

6616000 Supplementary Material
