# Supplementary material for: Multiple variants of tick-borne encephalitis virus in voles, mice and ticks, the Netherlands, 2021 to 2023
Source: Euro Surveill. 2025 Jan 30;30(4):2400247. doi: 10.2807/1560-7917.ES.2025.30.4.2400247 (PMC11920782; doi:10.2807/1560-7917.ES.2025.30.4.2400247)
Supplement: Supplementary Material [file 24-00247_PASCOE_Supplement.pdf]

Multiple variants of tick-borne encephalitis virus in voles, mice and ticks, the Netherlands, 2021 to 2023: Supplementary Materials

This supplementary material is hosted by Eurosurveillance as supporting information alongside the article “Multiple variants of tick-borne encephalitis virus in voles, mice and ticks, the Netherlands, 2021 to 2023”, on behalf of the authors, who remain responsible for the accuracy and appropriateness of the content. The same standards for ethics, copyright, attributions and permissions as for the article apply. Supplements are not edited by Eurosurveillance and the journal is not responsible for the maintenance of any links or email addresses provided therein.

Table S1. Summary of the main vegetation structure of three plots at three locations sampled in a survey of tick-borne encephalitis virus (TBEV), the Netherlands, June to September 2021. A characteristic photograph of the vegetation is presented, and any small but notable deviations from the overall plot description or image are described for each trapping grid therein. Trapping grids shaded in grey indicate those from which a TBEV-positive sample was collected.

**<u>Dronten</u>**			
**Plot D1**<br>Grid A, B, C, & D: Dense, mixed, broadleaf and coniferous forest, e.g., pedunculate oaks (*Quercus robur*), silver birch (*Betula pendula*), European lime (*Tilia × europaea*), sycamore maple (*Acer pseudoplatanus*), mountain-ash (*Sorbus aucuparia*) and *Prunus* spp. A few European black pine (*Pinus nigra*) struggling to survive. Dense understory of brambles and ferns.<br>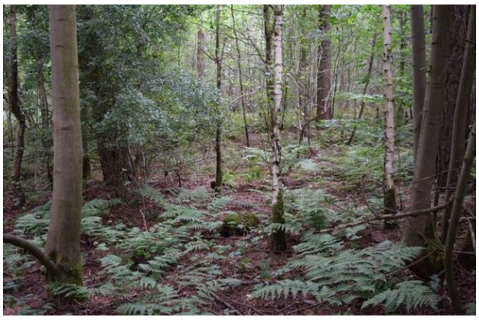			
A		B	No live pines; all dead.<br>Understory: Fewer brambles, more ferns.
C		D	Forest slightly more open (less dense).
**Plot D2**<br>Grid A, B, C, & D: Mixed broadleaf and coniferous forest, e.g., silver birch (*Betula pendula*), European black pine (*Pinus nigra*), sycamore maple (*Acer pseudoplatanus*), mountain-ash (*Sorbus aucuparia*) and *Prunus* spp.			

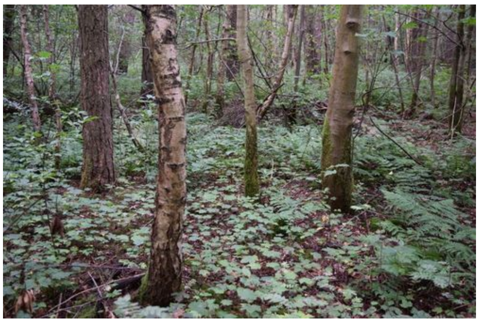

A		B	Understory: Dominated by ferns.
C		D	

Plot D3

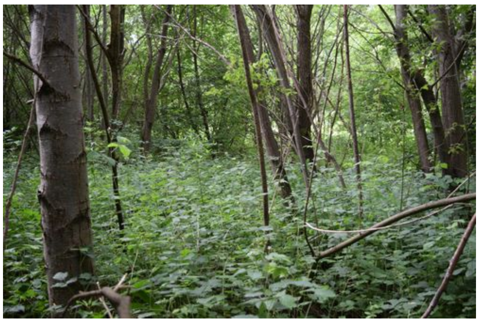

Grid A & B: Wet forest with goat willow (*Salix caprea*) dominant. Scattered common hazel (*Corylus avellana*), and sycamore maple (*Acer pseudoplatanus*).

Grid C & D: Coppiced common hazel.

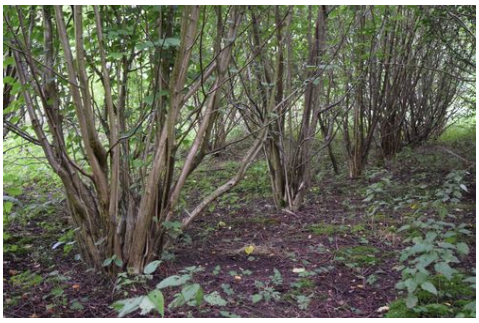

A	Understory: Thick brambles and ferns.	B	Scattered black elm (*Alnus glutinosa*), common lime (*Tilia × europaea*), and maythorn (*Crataegus spp.*).<br>Understory: Saplings

<table>
<tr><td>C</td><td>Understory: Brambles, few ferns.</td><td>D</td><td>Understory: Nettle</td></tr>
<tr><td colspan="4"><u>Zeist</u></td></tr>
<tr><td colspan="4">Plot Z1
Grid A, B, C, & D: Thinned stand of Scots pine (Pinus sylvestris), lower layer of scattered pedunculate oaks (Quercus robur), silver birch (Betula pendula) and mountain-ash (Sorbus aucuparia). Understory of blueberries, brambles and grasses.
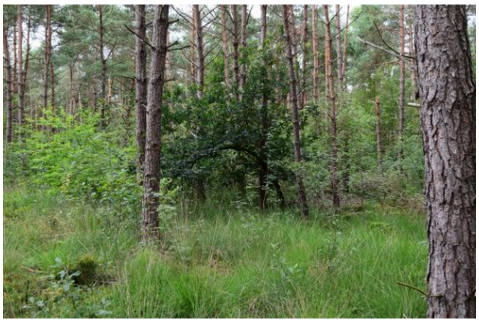</td></tr>
<tr><td>A</td><td></td><td>B</td><td></td></tr>
<tr><td>C</td><td>Lower layer dominated by silver birch (Betula pendula).
Understory: dominated by ferns.</td><td>D</td><td>Lower layer dominated by silver birch (Betula pendula).</td></tr>
<tr><td colspan="4">Plot Z2
Grid A & B: Dense, mixed broadleaf and coniferous forest, e.g., a few big (20 – 25 m) Scots pine (Pinus sylvestris), common buckthorn (Rhamnus frangula) dominant, Prunus spp. Understory of scattered bilberry (Vaccinium myrtillus).
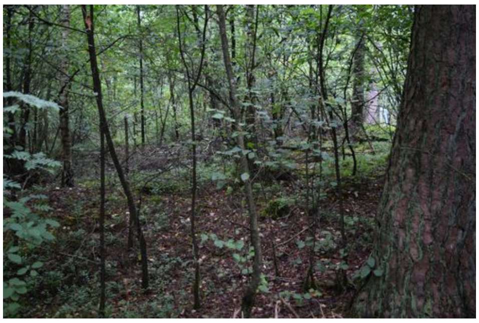
Grid C & D: Few large Scots pine (Pinus sylvestris), dense regeneration of Scots pine and silver birch (Betula pendula) (~5 – 6 years).</td></tr>
</table>

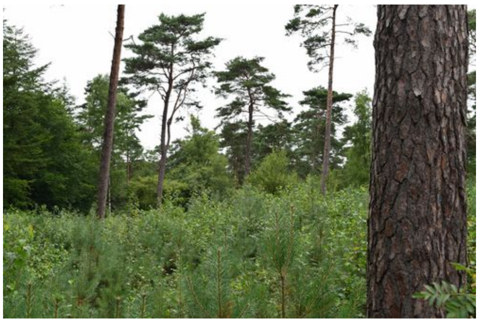

A		B	
C		D	

Plot Z3

Grid A & B: Very open old stand of Douglas fir (*Pseudotsuga menziesii*), dense lower layer of silver birch (*Betula pendula*), common hawthorn (*Crataegus monogyna*), mountain-ash (*Sorbus aucuparia*) and Scots pine (*Pinus sylvestris*).

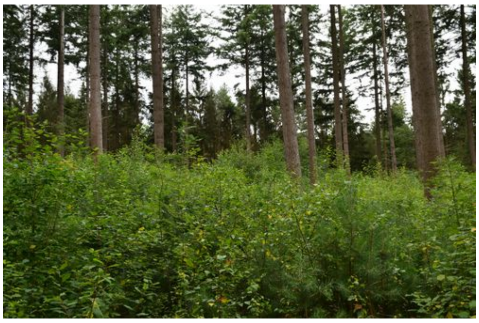

Grid C & D: Norway spruce dominant (*Picea abies*), scattered maythorn (*Crataegus spp.*), mountain-ash (*Sorbus aucuparia*), *Prunus* spp. and Scots pine (*Pinus sylvestris*). Understory of bilberry (*Vaccinium myrtillus*).

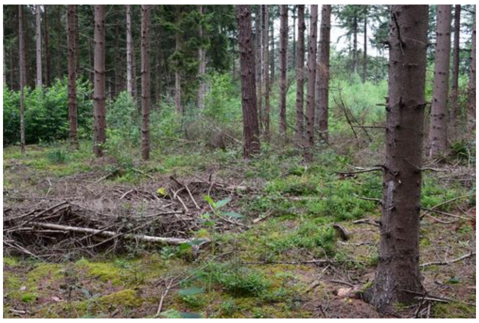

A		B	

<table>
<tr><td>C</td><td></td><td>D</td><td>Sparse understory.</td></tr>
<tr><td colspan="4"><u>Oost Gelre</u></td></tr>
<tr><td colspan="4">Plot L1
Grid A, B, C, & D: Pedunculate oak dominant forest (Quercus robur), with scattered silver birch (Betula pendula), mountain-ash (Sorbus aucuparia), Canadian poplar (Populus × canadensis), and Scots pine (Pinus sylvestris). Understory of brambles.
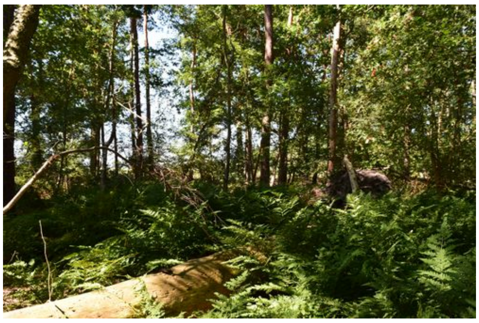</td></tr>
<tr><td>A</td><td></td><td>B</td><td></td></tr>
<tr><td>C</td><td>Forest slightly more open (less dense).</td><td>D</td><td>Understory: More fern and fewer brambles.</td></tr>
<tr><td colspan="4">Plot L2
Grid A & C: Mixed broadleaf forest of silver birch (Betula pendula), alder (Alnus glutinosa), mountain-ash (Sorbus aucuparia) and pedunculate oak (Quercus robur). Understory of brambles.
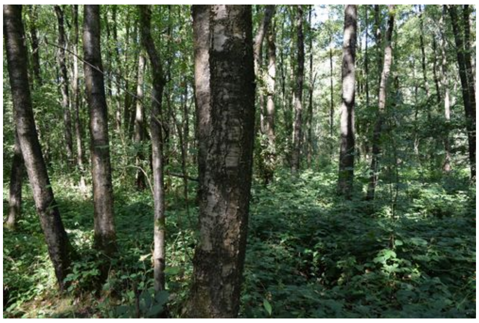
Grid B & D: Open, mixed broadleaf and coniferous forest. Pedunculate oak (Quercus robur) and silver birch (Betula pendula) somewhat dominant, with mountain-ash (Sorbus aucuparia) and Scots pine (Pinus sylvestris). Understory of brambles, fern and grasses.</td></tr>
</table>

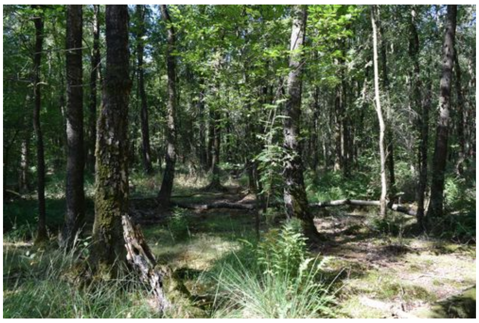

A		B	Understory: Also moss
C	Forest more open (less dense).<br>Understory: Also grasses	D	

Plot L3

Grid A & B: Young, planted oak and goat willow (*Salix caprea*) forest (~5 years maximum).

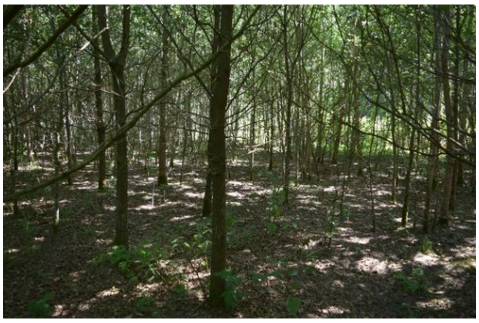

Grid C: Transect along edge of mixed forest dominated by pedunculate oak (*Quercus robur*), but with scattered European beech (*Fagus sylvatica*), silver birch (*Betula pendula*), common hazel (*Corylus avellana*), mountain-ash (*Sorbus aucuparia*). Mixed understory.

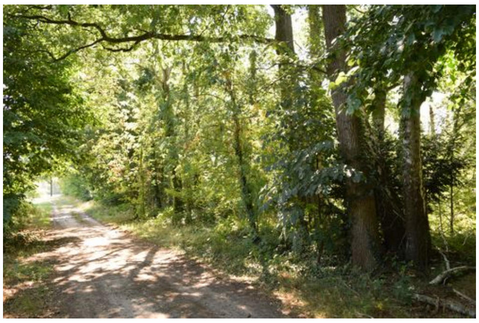

Grid D: Mixed forest dominated by pedunculate oak (*Quercus robur*) and silver birch

<table>
<tr><td colspan="4">(Betula pendula), with European beech (Fagus sylvatica) and mountain ash (Sorbus aucuparia). Understory of brambles.
Surrounded by bodies of water.
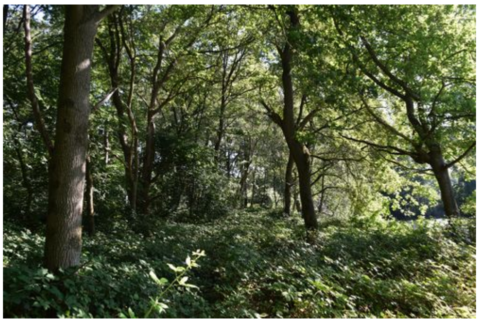</td></tr>
<tr><td>A</td><td>Trees a few years older.</td><td>B</td><td></td></tr>
<tr><td>C</td><td></td><td>D</td><td></td></tr>
</table>

Table S2: Overview of questing ticks grouped in pools for testing for tick-borne encephalitis virus (TBEV), by tick pool and tick stage, for a TBEV survey in the Netherlands, June to September 2021. Ticks were pooled according to life stage and transect, and tested for TBEV.

Pool ID	Location	Plot	Date collected	Larvae	Nymphs	Adult females	Adult males
1	Zeist	Z3	2021-09-16	0	0	1	0
2	Zeist	Z3	2021-09-16	0	0	0	1
3	Zeist	Z3	2021-09-16	0	7	0	0
4	Dronten	D2	2021-09-20	0	10*	0	0
5	Dronten	D2	2021-09-20	1	0	0	0
6	Dronten	D3	2021-09-20	0	1	0	0
7	Dronten	D2	2021-09-20	0	9	0	0
8	Oost Gelre	L2	2021-09-14	0	0	4	0
9	Oost Gelre	L2	2021-09-14	0	10	0	0
10	Oost Gelre	L2	2021-09-14	0	10	0	0
11	Oost Gelre	L2	2021-09-14	0	10	0	0
12	Oost Gelre	L2	2021-09-14	0	10	0	0
13	Oost Gelre	L2	2021-09-14	0	9	0	0
14	Oost Gelre	L2	2021-09-14	3	0	0	0
15	Zeist	Z1	2021-09-16	0	0	3	0
16	Zeist	Z1	2021-09-16	0	1	0	0
17	Zeist	Z1	2021-09-16	0	2	0	0
18	Zeist	Z2	2021-09-16	0	0	5	0
19	Zeist	Z2	2021-09-16	0	0	0	1
20	Zeist	Z2	2021-09-16	0	10	0	0
21	Zeist	Z2	2021-09-16	0	7	0	0
22	Oost Gelre	L1	2021-09-14	0	0	0	5
23	Oost Gelre	L1	2021-09-14	0	0	2	0
24	Oost Gelre	L1	2021-09-14	0	0	0	1
25	Oost Gelre	L1	2021-09-14	0	10	0	0
26	Oost Gelre	L1	2021-09-14	0	4	0	0
27	Oost Gelre	L3	2021-09-14	0	6	0	0
28	Dronten	D2	2021-09-20	0	10	0	0

29	Dronten	D2	2021-09-20	0	10	0	0
30	Dronten	D2	2021-09-20	0	0	0	3
31	Dronten	D2	2021-09-20	0	3	0	0
32	Dronten	D2	2021-09-20	4	0	0	0
33	Zeist	Z3	2021-07-05	0	4	0	0
34	Zeist	Z3	2021-07-05	0	0	1	0
35	Zeist	Z3	2021-07-05	0	0	0	1
36	Zeist	Z2	2021-07-05	0	10	0	0
37	Zeist	Z2	2021-07-05	0	10	0	0
38	Zeist	Z2	2021-07-05	0	10	0	0
39	Zeist	Z2	2021-07-05	0	10	0	0
40	Zeist	Z2	2021-07-05	0	10	0	0
41	Zeist	Z2	2021-07-05	0	0	5	0
42	Zeist	Z2	2021-07-05	0	10	0	0
43	Zeist	Z2	2021-07-05	0	10	0	0
44	Zeist	Z2	2021-07-05	0	10	0	0
45	Zeist	Z2	2021-07-05	0	10	0	0
46	Zeist	Z2	2021-07-05	0	11	0	0
47	Zeist	Z2	2021-07-05	0	0	0	4
48	Zeist	Z2	2021-07-05	0	0	1	0
49	Oost Gelre	L2	2021-08-16	0	10	0	0
50	Oost Gelre	L2	2021-08-16	0	7	0	0
51	Oost Gelre	L2	2021-08-16	0	0	1	0
52	Oost Gelre	L2	2021-08-16	0	0	0	1
53	Oost Gelre	L1	2021-08-09	0	0	5	0
54	Oost Gelre	L1	2021-08-09	0	0	0	5
55	Oost Gelre	L1	2021-08-09	0	0	4	0
56	Oost Gelre	L1	2021-08-09	0	0	0	4
57	Oost Gelre	L1	2021-08-09	0	10	0	0
58	Zeist	Z2	2021-07-05	0	0	5	0
59	Zeist	Z2	2021-07-05	0	10	0	0
60	Zeist	Z2	2021-07-05	0	10	0	0
61	Zeist	Z2	2021-07-05	0	10	0	0
62	Zeist	Z2	2021-07-05	0	10	0	0
63	Zeist	Z2	2021-07-05	0	0	4	0

64	Zeist	Z2	2021-07-05	0	0	0	3
65	Zeist	Z2	2021-07-05	0	10	0	0
66	Zeist	Z2	2021-07-05	0	6	0	0
67	Zeist	Z2	2021-07-05	0	10	0	0
68	Zeist	Z2	2021-07-05	0	10	0	0
69	Zeist	Z2	2021-07-05	0	10	0	0
70	Zeist	Z2	2021-07-05	0	10	0	0
71	Zeist	Z2	2021-07-05	0	7	0	0
72	Zeist	Z2	2021-07-05	0	0	1	0
73	Zeist	Z3	2021-07-05	0	1	0	0
74	Oost Gelre	L1	2021-08-09	0	0	1	0
75	Oost Gelre	L1	2021-08-09	0	5	0	0
76	Oost Gelre	L3	2021-08-23	0	13	0	0
77	Oost Gelre	L3	2021-08-23	1	0	0	0
78	Oost Gelre	L3	2021-08-23	0	0	0	1
79	Dronten	D3	2021-06-15	0	10	0	0
80	Dronten	D3	2021-06-15	0	0	5	0
81	Dronten	D3	2021-06-15	0	0	0	5
82	Dronten	D3	2021-06-15	0	10	0	0
83	Dronten	D3	2021-06-15	0	0	0	5
84	Dronten	D3	2021-06-15	0	14	0	0
85	Dronten	D3	2021-06-15	0	0	0	1
86	Zeist	Z1	2021-06-25	0	3	0	0
87	Zeist	Z1	2021-06-25	0	10	0	0
88	Zeist	Z1	2021-06-25	0	10	0	0
89	Zeist	Z1	2021-06-25	0	0	2	0
90	Zeist	Z1	2021-06-25	0	0	0	1
91	Zeist	Z1	2021-06-25	0	10	0	0
92	Zeist	Z1	2021-06-25	0	10	0	0
93	Zeist	Z1	2021-06-25	0	0	5	0
94	Zeist	Z1	2021-06-25	0	0	0	5
95	Dronten	D3	2021-06-15	0	0	2	0
96	Dronten	D3	2021-06-15	0	0	0	4
97	Dronten	D3	2021-06-15	0	5	0	0

98	Zeist	Z1	2021-06-25	0	10	0	0
99	Zeist	Z1	2021-06-25	0	10	0	0
100	Zeist	Z1	2021-06-25	0	10	0	0
101	Zeist	Z1	2021-06-25	0	10	0	0
102[†]	Zeist	Z1	2021-06-25	1	9	0	0
103	Zeist	Z1	2021-06-25	0	0	0	2
104	Dronten	D2	2021-06-15	0	10	0	0
105	Dronten	D2	2021-06-15	0	10	0	0
106	Dronten	D2	2021-06-15	0	10	0	0
107	Dronten	D2	2021-06-15	0	10	0	0
108	Dronten	D2	2021-06-15	10	0	0	0
109	Dronten	D2	2021-06-15	10	0	0	0
110	Dronten	D2	2021-06-15	0	0	0	5
111	Dronten	D2	2021-06-15	0	10	0	0
112	Dronten	D2	2021-06-15	10	0	0	0
113	Dronten	D2	2021-06-15	0	10	0	0
114	Dronten	D2	2021-06-15	10	0	0	0
115	Dronten	D2	2021-06-15	4	0	0	0
116	Dronten	D2	2021-06-15	0	0	1	0
117	Dronten	D2	2021-06-15	0	0	5	0
118	Dronten	D2	2021-06-15	0	10	0	0
119	Dronten	D2	2021-06-15	0	0	4	0
120	Dronten	D2	2021-06-15	0	5	0	0
121	Dronten	D2	2021-06-15	0	0	0	3
122	Zeist	Z1	2021-06-25	0	10	0	0
123	Zeist	Z1	2021-06-25	0	10	0	0
124	Zeist	Z1	2021-06-25	0	9	0	0
125	Zeist	Z1	2021-06-25	0	0	3	0
126	Zeist	Z1	2021-06-25	0	0	0	1
127	Dronten	D2	2021-06-15	0	10	0	0
128	Dronten	D2	2021-06-15	10	0	0	0
129	Dronten	D2	2021-06-15	0	10	0	0
130	Dronten	D2	2021-06-15	0	10	0	0
131	Dronten	D2	2021-06-15	10	0	0	0

132	Dronten	D2	2021-06-15	10	0	0	0
133	Dronten	D2	2021-06-15	0	10	0	0
134	Dronten	D2	2021-06-15	0	10	0	0
135	Dronten	D2	2021-06-15	10	0	0	0
136	Dronten	D2	2021-06-15	0	10	0	0
137	Dronten	D2	2021-06-15	10	0	0	0
138	Dronten	D2	2021-06-15	0	10	0	0
139	Dronten	D2	2021-06-15	0	0	0	4
140	Dronten	D2	2021-06-15	0	0	2	0
141	Dronten	D2	2021-06-15	10	0	0	0
142	Dronten	D2	2021-06-15	10	0	0	0
143	Dronten	D2	2021-06-15	10	0	0	0
144	Dronten	D2	2021-06-15	6	0	0	0
145	Dronten	D2	2021-06-15	0	11	0	0
146	Dronten	D2	2021-06-15	0	10	0	0
147	Dronten	D2	2021-06-15	0	10	0	0
148	Dronten	D2	2021-06-15	0	10	0	0
149	Dronten	D2	2021-06-15	0	10	0	0
150	Dronten	D2	2021-06-15	0	10	0	0
151	Dronten	D2	2021-06-15	0	10	0	0
152	Dronten	D2	2021-06-15	0	0	1	0
153	Dronten	D2	2021-06-15	0	10	0	0
154	Dronten	D2	2021-06-15	0	4	0	0
155	Dronten	D2	2021-06-15	10	0	0	0
156	Dronten	D1	2021-06-02	0	10	0	0
157	Dronten	D1	2021-06-02	0	10	0	0
158	Dronten	D1	2021-06-02	0	10	0	0
159	Dronten	D1	2021-06-02	0	10	0	0
160	Dronten	D1	2021-06-02	0	0	3	0
161	Dronten	D1	2021-06-02	0	10	0	0
162	Dronten	D1	2021-06-02	0	10	0	0
163	Dronten	D1	2021-06-02	0	0	0	3
164	Dronten	D1	2021-06-02	0	10	0	0
165	Dronten	D1	2021-06-02	0	10	0	0

166	Dronten	D1	2021-06-02	0	10	0	0
167	Dronten	D1	2021-06-02	0	10	0	0
168	Dronten	D1	2021-06-02	0	10	0	0
169	Dronten	D1	2021-06-02	0	10	0	0
170	Dronten	D1	2021-06-02	0	10	0	0
171	Dronten	D1	2021-06-02	0	10	0	0
172	Dronten	D1	2021-06-02	10	0	0	0
173	Dronten	D1	2021-06-02	0	10	0	0
174	Dronten	D1	2021-06-02	10	0	0	0
175	Dronten	D1	2021-06-02	0	0	0	2
176	Dronten	D1	2021-06-02	7	0	0	0
177	Dronten	D1	2021-06-02	0	6	0	0
178	Dronten	D3	2021-06-15	0	10	0	0
179	Dronten	D3	2021-06-15	0	10	0	0
180	Dronten	D3	2021-06-15	10	0	0	0
181	Dronten	D3	2021-06-15	10	0	0	0
182	Dronten	D3	2021-06-15	0	10[*,S]	0	0
183	Dronten	D3	2021-06-15	0	0	0	5
184	Dronten	D3	2021-06-15	10	0	0	0
185	Dronten	D3	2021-06-15	10	0	0	0
186	Dronten	D3	2021-06-15	10	0	0	0
187	Dronten	D3	2021-06-15	0	11	0	0
188	Dronten	D3	2021-06-15	16	0	0	0
189	Dronten	D3	2021-06-15	0	0	2	0
190	Dronten	D3	2021-06-15	0	0	0	1
191	Dronten	D1	2021-06-02	0	10	0	0
192	Dronten	D1	2021-06-02	0	0	5	0
193	Dronten	D1	2021-06-02	0	10	0	0
194	Dronten	D1	2021-06-02	0	10	0	0
195	Dronten	D1	2021-06-02	0	10	0	0
196	Dronten	D1	2021-06-02	0	10	0	0
197	Dronten	D1	2021-06-02	0	0	0	5
198	Dronten	D1	2021-06-02	0	11	0	0
199	Dronten	D1	2021-06-02	0	10	0	0

200	Dronten	D1	2021-06-02	0	0	0	3
201	Dronten	D1	2021-06-02	0	0	1	0
202	Dronten	D1	2021-06-02	0	10	0	0
203	Dronten	D1	2021-06-02	0	10	0	0
204	Dronten	D1	2021-06-02	0	10	0	0
205	Dronten	D1	2021-06-02	0	10	0	0
206	Dronten	D1	2021-06-02	0	10	0	0
207	Dronten	D1	2021-06-02	0	0	0	5
208	Dronten	D1	2021-06-02	0	10	0	0
209	Dronten	D1	2021-06-02	0	0	5	0
210	Dronten	D1	2021-06-02	0	10	0	0
211	Dronten	D1	2021-06-02	0	10	0	0
212	Dronten	D1	2021-06-02	0	0	0	2
213	Dronten	D1	2021-09-20	0	11	0	0
214	Dronten	D1	2021-09-20	0	0	2	0
215	Dronten	D1	2021-09-20	0	0	0	3

[*] = tick pool positive for TBEV-RNA; [S] = tick pool sequenced; [†] = tick pool contained multiple life stages

Table S3. Overview of biometric characteristics of rodents (n = 383) captured in a survey of tick-borne encephalitis virus (TBEV), by three sampled locations, the Netherlands, June to August 2021.

	**Dronten**				**Zeist**				**Oost Gelre**			
	**D1 (n=35)**	**D2 (n=30)**	**D3 (n=51)**	**Total (n=116)**	**Z1 (n=31)**	**Z2 (n=36)**	**Z3 (n=52)**	**Total (n=119)**	**L1 (n=63)**	**L2 (n=48)**	**L3 (n=37)**	**Total (n=148)**
**Sex**												
Female	17/35	16/30	24/51	**49.1%**	11/31	13/36	26/52	**42.0%**	35/63	21/48	19/37	**50.7%**
Male	17/35	14/30	22/51	**45.7%**	19/31	14/36	18/52	**42.9%**	25/63	21/48	16/37	**41.9%**
Unrecorded	1/35	0/30	5/51	**5.2%**	1/31	9/36	8/52	**15.1%**	3/63	6/48	2/37	**7.4%**
**Breeding status**												
Adult	20/35	28/30	20/51	**58.6%**	19/31	14/36	31/52	**53.8%**	40/63	26/48	30/37	**64.9%**
Subadult	0/35	0/30	4/51	**3.4%**	7/31	11/36	5/52	**19.3%**	0/63	0/48	0/37	**0%**
Juvenile	15/35	2/30	14/51	**26.7%**	5/31	2/36	9/52	**13.4%**	19/63	8/48	3/37	**20.3%**
Unrecorded	0/35	0/30	13/51	**11.2%**	0/31	9/36	7/52	**13.4%**	4/63	14/48	4/37	**14.9%**
**Tick burdens**												
No. rodents checked for ticks	0	15	20	**35**	31	26	18	**75**	39	27	29	**95**
Percent of rodents with ticks	na	15/15	17/20	**91.4%**	31/31	25/26	18/18	**98.7%**	28/39	19/27	23/29	**73.7%**
Median no. of	na	57	14	**23**	28	11	10	**20**	3	4	4	**4**

larvae (range)		(6–130)	(0–110)	**(0–130)**	(2–95)	(0–50)	(8–40)	**(0–95)**	(0–30)	(0–40)	(0–35)	**(0–40)**
Percent of rodents with nymphs (burden range)	na	1/15 na	3/20 (1–4)	**11.4% (1–4)**	15/31 (1–4)	2/26 (2)	1/18 (2)	**24.0% (1–4)**	1/39 (1)	2/27 (1)	2/29 (1–2)	**5.3% (1–2)**

Table S4: Overview of tick-borne encephalitis virus (TBEV) sequences used in a Monte Carlo simulation method to test for TBEV-variant over-representation in European countries. Variant identity based on clustering by CD-HIT using a 99% similarity threshold is shown.

GenBank® accession/sequence ID	Country	Variant ID
KF151173	Austria	4
MT311861	Austria	44
MG243699	Austria	57
KJ922514	Czechia	30
KJ922515	Czechia	34
KJ922513	Czechia	34
DQ153877	Czechia	38
KJ922516	Czechia	49
KJ922512	Czechia	55
MN735990	Denmark	53
MN735989	Denmark	53
MN735988	Denmark	53
MN735991	Denmark	92
OQ435379	Estonia	14
GU183383	Estonia	23
MH094241	Estonia	43
DQ486861	Estonia	43
GU183384	Estonia	97
KT224357	Finland	6
GU183380	Finland	6
GU183379	Finland	6
MK801814	Finland	14
MK801813	Finland	14
MK801812	Finland	14
MK801811	Finland	14
MK801810	Finland	14
MK801809	Finland	14
MG589938	Finland	14
MG589937	Finland	14

MK801804	Finland	27
MK801803	Finland	31
MK801808	Finland	33
MK801806	Finland	33
MK801805	Finland	33
MK801807	Finland	63
GU183381	Finland	98
MN047455	Finland	117
OL441148	France	40
KX268728	Germany	3
GQ266392	Germany	9
FJ572210	Germany	16
MK922617	Germany	19
MK922616	Germany	19
MK922615	Germany	19
KU884607	Germany	20
AM600965	Germany	46
MW256716	Hungary	29
MG210948	Hungary	29
MG210947	Hungary	29
MG210946	Hungary	29
MG210945	Hungary	29
MW256715	Hungary	50
OM084948	Italy	9
GU183382	Latvia	42
DZIF23_597	Netherlands	18
LC811643	Netherlands	8
MZ969636	Netherlands	8
MH021184	Netherlands	8
LC811640	Netherlands	19
MZ969639	Netherlands	19
MZ969638	Netherlands	19
LC811644	Netherlands	23
LC811645	Netherlands	41
ON502378	Netherlands	41

LC171402	Netherlands	41
LC811642	Netherlands	72
LC811641	Netherlands	85
KF991107	Norway	53
KC835595	Slovakia	1
KC835597	Slovakia	5
KC835596	Slovakia	32
ON228410	Slovenia	10
OQ889223	Slovenia	11
JQ654701	Slovenia	11
OQ889253	Slovenia	12
OQ889251	Slovenia	12
ON228429	Slovenia	17
OQ889256	Slovenia	21
OQ889250	Slovenia	21
OQ889246	Slovenia	21
OQ889239	Slovenia	21
ON228428	Slovenia	21
ON228430	Slovenia	24
ON228434	Slovenia	25
ON228432	Slovenia	25
OQ889170	Slovenia	26
OQ889175	Slovenia	28
OQ889171	Slovenia	28
OQ889190	Slovenia	35
OQ889185	Slovenia	36
ON228412	Slovenia	37
OQ889200	Slovenia	39
OQ889240	Slovenia	45
OQ889204	Slovenia	47
OQ889242	Slovenia	48
OQ889245	Slovenia	51
ON228414	Slovenia	51

OQ889224	Slovenia	52
OQ889226	Slovenia	54
OQ889234	Slovenia	56
ON228415	Slovenia	58
OQ889233	Slovenia	59
OQ889199	Slovenia	60
OQ889176	Slovenia	61
ON228416	Slovenia	62
ON228417	Slovenia	64
OQ889247	Slovenia	65
OQ889177	Slovenia	66
ON228418	Slovenia	67
OQ889208	Slovenia	68
OQ889191	Slovenia	69
OQ889252	Slovenia	70
OQ889172	Slovenia	71
ON228413	Slovenia	73
ON228419	Slovenia	74
OQ889203	Slovenia	75
OQ889220	Slovenia	76
ON228420	Slovenia	77
OQ889184	Slovenia	78
OQ889227	Slovenia	79
ON228421	Slovenia	80
OQ889201	Slovenia	81
OQ889209	Slovenia	82
OQ889188	Slovenia	83
OQ889182	Slovenia	84
OQ889210	Slovenia	86
OQ889221	Slovenia	87
OQ889205	Slovenia	89
OQ889183	Slovenia	90
OQ889232	Slovenia	91
OQ889225	Slovenia	93
ON228422	Slovenia	94

OQ889213	Slovenia	95
OQ889214	Slovenia	96
OQ889186	Slovenia	99
OQ889187	Slovenia	100
OQ889217	Slovenia	101
OQ889168	Slovenia	102
ON228423	Slovenia	103
OQ889192	Slovenia	104
OQ889218	Slovenia	105
OQ889173	Slovenia	106
OQ889196	Slovenia	107
OQ889167	Slovenia	108
OQ889206	Slovenia	109
OQ889193	Slovenia	110
OQ889207	Slovenia	111
OQ889228	Slovenia	112
ON228425	Slovenia	113
ON228433	Slovenia	114
OQ889229	Slovenia	115
OQ889211	Slovenia	116
ON228431	Slovenia	118
OQ889243	Slovenia	119
OQ889197	Slovenia	120
OQ889189	Slovenia	121
ON228427	Slovenia	122
ON228426	Slovenia	123
ON228424	Slovenia	124
OQ889202	Slovenia	125
OQ889178	Slovenia	126
OQ889248	Slovenia	127
OQ889216	Slovenia	129
OQ889238	Slovenia	130
OQ889169	Slovenia	131

OQ889230	Slovenia	132
ON228411	Slovenia	133
OQ889194	Slovenia	134
OQ889241	Slovenia	135
OQ889195	Slovenia	136
OQ889237	Slovenia	137
OQ889212	Slovenia	138
OQ889215	Slovenia	139
OQ889231	Slovenia	140
OQ889181	Slovenia	141
OQ889198	Slovenia	142
OQ889219	Slovenia	143
OQ889174	Slovenia	144
OQ889244	Slovenia	145
OQ889255	Slovenia	146
OQ889235	Slovenia	147
OQ889222	Slovenia	148
OQ889179	Slovenia	149
OQ889180	Slovenia	150
OQ889254	Slovenia	151
OQ889236	Slovenia	152
OQ889249	Slovenia	153
ON228409	Slovenia	154
ON228408	Slovenia	154
KX966398	Sweden	7
DQ401140	Sweden	7
MT581212	Sweden	8
MT311860	Sweden	8
KF991106	Sweden	13
KX966399	Sweden	15
OR523238	Switzerland	2
OQ555314	Switzerland	10
OQ555315	Switzerland	22
OQ555317	Switzerland	88
OQ555316	Switzerland	128

MN661145	United Kingdom	41
MN128700	United Kingdom	53

Table S5: Results of a Monte Carlo simulation method to test for over-representation of tick-borne encephalitis virus-variants in European countries. Variant identity was based on clustering by CD-HIT using a 99% similarity threshold. A p-value ≤ 0.05 indicated that the observed number of variants for a country was lower than expected by chance, and therefore over-representation of one or more TBEV-variants, while $p > 0.05$ suggested that the observed number of TBEV-variants in the country did not deviate from random simulations.

Country	No. sequences	No. variants	***p*-value**
Austria	3	3	1
Czechia	6	5	0.46
Denmark	4	2	0.01
Estonia	5	4	0.33
Finland	19	8	<0.01
France	1	1	1
Germany	8	6	0.23
Hungary	6	2	<0.01
Italy	1	1	1
Latvia	1	1	1
The Netherlands	13	7	0.01
Norway	1	1	1
Slovakia	3	3	1
Slovenia	118	108	1
Sweden	6	4	0.07
Switzerland	5	5	1
United Kingdom	2	2	1
